# From Concept to Practice: Using the School Health Index to Create Healthy School Environments in Rhode Island Elementary Schools

**Published:** 2005-10-15

**Authors:** Deborah N Pearlman, Elizabeth Dowling, Cheryl Bayuk, Kathleen Cullinen, Ann Kelsey Thacher

**Affiliations:** Rhode Island Department of Health, Division of Disease Prevention and Control; Dr. Pearlman is also Assistant Professor of Community Health (Research), Department of Community Health, Brown University, Providence, RI.; Senior Epidemiologist, Arkansas Department of Health and Human Services, Assistant Professor, University of Arkansas for Medical Sciences College of Public Health, Little Rock, Ark; Rhode Island Department of Health, Providence, RI; Rhode Island Department of Health, Providence, RI; Rhode Island Department of Health, Providence, RI

## Abstract

**Introduction:**

The prevalence of childhood obesity is increasing, and schools are ideal places to support healthy eating and physical activity. In 2000, the Centers for Disease Control and Prevention (CDC) developed the School Health Index, a self-assessment and planning tool that helps schools evaluate and improve physical activity and nutrition programs and policies*.* Although many state education agencies, health departments, and individual schools have used the School Health Index, few systematic evaluations of the tool have been performed. We examined the physical activity and nutrition environments in Rhode Island's public elementary schools with high and low minority student enrollments and evaluated a school-based environmental and policy intervention that included implementation of the School Health Index.

**Methods:**

As part of a CDC Division of Nutrition and Physical Activity cooperative agreement awarded to the Rhode Island Department of Health, we conducted a needs assessment of 102 elementary schools and implemented an intervention in four inner-city elementary schools. In phase 1, we analyzed the Rhode Island Needs Assessment Tool (RINAT), a telephone survey of principals in approximately 50% of all Rhode Island public elementary schools in the state during the 2001–2002 school year (n = 102). Comparisons of the nutrition and physical activity environments of schools with low and high minority enrollment were calculated by cross-tabulation with the chi-square test. In phase 2, we used process and outcome evaluation data to assess the use of the School Health Index in creating healthier environments in schools. Our intervention — *Eat Healthy and Get Active! *— involved implementing three of the eight School Health Index modules in four Rhode Island elementary schools.

**Results:**

Survey data revealed that schools with high minority enrollment (student enrollment of ≥10% black, ≥25% Hispanic, or both) offered few programs supporting healthy eating and physical activity (*P* < .05). Schools with high and low minority enrollment both offered nonnutritious foods and beverages. Process evaluation data revealed that 1) principals play a pivotal role on School Health Index teams, 2) schoolwide validation of a team's small successes is crucial for sustaining a commitment to healthy lifestyle policies and programs, and 3) external facilitators are essential for implementation success. Outcome data showed that all schools developed at least one policy or environmental strategy to create a healthy school environment. Only two schools implemented immediate changes.

**Conclusion:**

Needs assessment, external facilitation, and evaluation are the foundation for sustainable school-based policies. Although the School Health Index is universally perceived as a user-friendly assessment tool, implementation is likely to be less successful in schools with low staff morale, budgetary constraints, and inconsistent administrator support.

## Introduction

U.S. children today are more likely to be overweight than children in previous decades, and the upward trend in the prevalence of childhood obesity is continuing (1). Between 1980 and 2000, the prevalence of obesity doubled among children aged 6 to 11 years (2). The increase is particularly evident among non-Hispanic black and Hispanic youth (3).

Schools are ideal places to support healthy eating and physical activity (4). However, the 2000 national School Health Policies and Programs Study (SHPPS) found that only 8% of elementary schools provided daily physical education; 71.4% provided regular recess for elementary school children (5). In 43% of elementary schools, food and beverages of little or no nutritional value were readily available (5). Many national initiatives call for strengthening school-based policies and environments (6-12). In 2000, the Centers for Disease Control and Prevention (CDC) developed the School Health Index (SHI), consisting of eight modules drawn from the CDC's Coordinated School Health Program model. The model describes a healthy school environment as one in which the integration of policies, practices, and programs promote healthy lifestyle behaviors and reduce health-related risk behaviors (13). Using the SHI, teams composed of administrators, teachers, food service personnel, and other members of the school community assess the school's strengths and weaknesses in eight areas and then plan for improvement (14).

Many state education agencies, health departments, and individual schools have used the SHI, but systematic evaluations have been rare. Staten et al evaluated the SHI in seven elementary schools in two Arizona border communities (15). Although most schools implemented changes using the SHI, staff turnover, time constraints, and limited resources were barriers to progress (15). Schools do not exist in a vacuum; school-based programs and policies are likely to fail if the environment lacks the infrastructure to promote healthy eating and physical activity (12).

In our study, we address the following two questions:

1. What is the difference between the physical activity and nutrition environments in Rhode Island's public elementary schools with high minority student enrollment (≥10% black, ≥25% Hispanic, or both) and schools with low minority student enrollment (<10% black and <25% Hispanic)?
2. Does the SHI help schools create healthy school environments?

We describe the results of a needs assessment to understand the extent to which Rhode Island elementary schools promoted healthy eating and physical activity and had policies that supported these behaviors (phase 1). We also provide case studies of four elementary schools that participated in the *Eat Healthy and Get Active!* project, an intervention to help schools develop policies and environmental supports that promote lifelong physical activity and healthy eating (phase 2). In the intervention schools, 25% or more of the students were Hispanic or black, and the schools were located in cities with a relatively low tax base; thus, they had fewer resources for improving students' eating and physical activity behaviors. School personnel had become interested in healthier environments for their students in response to recent publicity about the increase in obesity among children, particularly among minorities (1,2).

## Methods

### Phase 1: needs assessment

The needs assessment involved three data sources: the 2001–2002 Rhode Island Needs Assessment Tool (RINAT), Rhode Island's 2002 *Information Works!* (16), and the 2000 U.S. census (17,18). *Information Works!* is a yearly report that contains detailed information on every public school and school district in Rhode Island, including standardized achievement scores, demographic information on students and parents, and data on school spending (16).

RINAT was designed by the Rhode Island Department of Health's Initiative for a Healthy Weight Program to assess environmental and policy support for healthy eating and physical activity in the state's elementary schools (Appendix). The sampling frame consisted of all public elementary schools in Rhode Island (N = 212). Recruitment was a two-step process. First, all elementary schools with a family center were selected. Schools with family centers receive federal and state funds to work with families in economically disadvantaged communities (19). Of the 35 family center schools, 32 completed interviews (91%). Of the remaining schools in Rhode Island, 100 were randomly selected for the study from five strata based on the telephone exchanges used for the Rhode Island Behavioral Risk Factor Surveillance System (BRFSS). Ninety-four schools from the second sampling frame were selected, and 70 completed interviews (75%). The overall response rate was 79%, and the final sample included 102 schools. School principals were interviewed by telephone between November 2001 and May 2002.

**Dependent variable**

From *Information Works!* data (16), we calculated the percentage of black and Hispanic students enrolled in Rhode Island public schools. To reflect the demographics of Rhode Island's overall student population, we defined a *high minority school* as one with a student population that was 10% black or greater, 25% Hispanic or greater, or both. In cities with the highest concentration of minority students, 8% to 23% of students were black, and 24% to 63% of students were Hispanic (16).

**Independent variables**

Because RINAT did not include questions on family demographics, we used *Information Works!* data to obtain the information (16). In 2002, 34% of Rhode Island elementary school students were eligible for free or reduced-price lunches, and 10% had one parent who did not complete high school (16). Therefore, the percentage of students eligible for free or reduced-price lunch was categorized as either *less than 34%* or *34% or greater,* and the percentage of students' parents who did not complete high school was categorized as *less than 5%* or *5% or greater* because of missing data on this variable.

RINAT variables were coded either *no* (no = 0) or *yes* (yes = 1). The variables included whether a school 1) had at least one program to promote healthy eating; 2) served high-fat or high-sugar foods in the cafeteria, vending machines, or other venues; 3) had one or more programs to promote physical activity; 4) had a playground, playing field, or track; 5) provided at least 20 minutes of recess per day; and 6) provided at least 60 minutes of physical education per week. In Rhode Island, no school met the National Association for Sport and Physical Education recommendation of 150 minutes per week of physical education for elementary schools (20). Therefore, we dichotomized responses on minutes of recess and minutes of physical education classes at the median.

Block-group census data were matched to each school based on the school's street address and zip code to measure residential racial segregation, an indicator of the socioeconomic status of the school's neighborhood. Literature on social inequalities has shown that residential racial segregation is the single most important factor in creating neighborhoods of concentrated poverty (21). To reflect the demographics of the three Rhode Island cities with the highest concentration of minority residents, we defined a racially segregated neighborhood as 10% black or greater, 15% Hispanic or greater, or both. In the three cities, 5.8% to 14.5% of residents were black, and 13.9% to 47.8% of residents were Hispanic (18).

### Phase 2: intervention and case studies

As mentioned previously, *Eat Healthy and Get Active!* was an intervention to help schools develop policies and environmental support that promote lifelong physical activity and healthy eating. Through a competitive process, the Rhode Island Department of Health selected the nonprofit Kids First, Inc to implement the intervention. From September 2002 through June 2003, four schools in three school districts participated in the project. Participating schools received $500 and training on use of the SHI manual.

*Eat Healthy and Get Active!* had five components. Each intervention school 1) established an SHI team, 2) completed three of the eight SHI self-assessment modules (the school policies and environment module, the physical education and other physical activity programs module, and the nutrition services module), 3) developed action plans to implement policies to improve students' physical activity and nutritious eating behaviors, 4) collected process and outcome data, and 5) worked with an external facilitator who provided continuity and resources. We focused only on the three SHI modules that included explicit policy recommendations. Getting a school board to approve and adopt policies was a longer-term process that would have exceeded our 10-month intervention.

The SHI was implemented differently in each school, but all schools established an SHI team and identified an internal coordinator for the intervention. All teams included the principal, a physical education teacher, and a food service director or manager. Although the intervention ran from September through June, it took until October to get school teams established.

**Process evaluation**

Process evaluation was designed to assess implementation of the intervention and external factors that may have affected the intervention's impact on study outcomes. Methods for monitoring implementation included evaluations of facilitator trainings on childhood obesity and use of the SHI, responses to discussion and planning questions from SHI modules, facilitator meeting notes and observations of team meetings, and pretest and posttest interviews with SHI team members. Process evaluation methods to monitor external factors affecting program implementation included an activity report form for tracking educational activities that were not part of the intervention and a form to record observations of the school environment, such as food pyramid pictures in the cafeteria, lunch time plate waste (the quantity of edible food served that is uneaten), and advertisements for fast foods within a 1-mile radius of the school. We observed each school's immediate neighborhood environment because the CDC's KidsWalk-to-School program (22) sets a 1-mile radius as ideal for walking to and from school by elementary school children. We highlight the process evaluation findings from facilitator meeting notes.

**Outcome evaluation**

The outcome measures were 1) baseline (October 2002) to end-of-year (June 2003) percent change in SHI self-assessment module scores and 2) the number of policies developed and implemented. The module score was the total number of points received for each question in a module (0 = not in place, 1 = under development, 2 = partially in place, 3 = fully in place) divided by the highest possible score for that module, then multiplied by 100.

**Statistical analysis**

Comparisons of the nutrition and physical activity environments of schools with low and high minority enrollment were calculated by cross-tabulation with the chi-square test (phase 1) using SPSS, version 12 (SPSS Inc, Chicago, Ill). Two external facilitators independently identified themes from the process evaluation, and the themes were ranked from most cited to least cited. *Eat Healthy and Get Active!* staff members reviewed the pooled results (phase 2). The Rhode Island Department of Health's Institutional Review Board approved both phases of the study.

## Results

### Phase 1: needs assessment

In our survey sample, 42.2% of the schools had a high minority student enrollment (Table 1). In schools in which 34% or more of the students were eligible for free or reduced-price lunch, 78.0% had a high minority student enrollment. In schools in which 5% or more of the students' parents did not have a high school diploma, 69.6% had a high minority student enrollment. Racially segregated neighborhoods were as likely as predominantly white communities to have schools with a high minority student enrollment, but our sample of racially segregated neighborhoods was small (n = 12), which limited our analyses.

Table 2 presents characteristics of the nutrition and physical activity environments in the participating schools. Schools with high minority student enrollment were less likely than those with low minority student enrollment to have programs promoting healthy eating or physical activity or have a playing field or track on the school grounds. In high minority schools, the time allotted for recess was an average of 27 minutes per day, whereas in low minority schools the recess time was an average of 16 minutes (data not shown). No differences between the two types of schools were found in the availability of nonnutritious foods and sweetened beverages that students could purchase during school hours. Only 10% of all schools reported having written policies on nutrition, physical activity, or both, excluding policies mandated by the state (data not shown).

### Phase 2: intervention and case studies

Table 3 shows the demographic characteristics of the four schools that participated in the intervention. The four schools combined included grades prekindergarten through 6: school 1 had grades prekindergarten and kindergarten; school 2, grades kindergarten and 1; school 3, grades kindergarten through 6; and school 4, grades 4 through 6. Student enrollments in the various schools ranged from 118 to 547 students, with Hispanic students comprising 26% to 65% of the student body. Student eligibility for free or reduced-price lunch ranged from 54% to 93%.

As mentioned, all teams included the principal, a physical education teacher, and a food service director or manager. Some teams had teachers, the school nurse, a parent, the president of the parent–teacher organization, the director from the school's family center, or all of these. In all, the principal played a pivotal role in team functioning. In three of the four schools, the principal's support was a key component to the success of the intervention. In the fourth school, existing tension between the principal and staff was a barrier.

Team size ranged from 5 to more than 20 members. Regardless of a school's team size, getting regular attendance at team meetings was challenging. The team size did not seem to affect implementation of strategies; for example, the largest team implemented the fewest strategies. What mattered more than the team size was the attitude of the team members; smaller teams with members who were enthusiastic, decisive, and proactive were able to accomplish more than larger teams with conflicting agendas. Teams had formal meetings from October through June. Three teams were subcommittees of the School Improvement Team, a team that is mandated for all schools by Rhode Island statute (23).

All schools completed the SHI assessment early in fall 2002. Team members were excited to implement changes. Three schools drafted action plans by the end of fall for implementation during spring 2003. The facilitators encouraged teams to address one action or policy at a time. Each policy had many steps that preceded full implementation. Through small successes, SHI teams recognized that they would have greater support if they recommended one proposal at a time rather than a long list of changes. It also became clear that some of the proposed changes would be difficult to implement, either because the policy required a lengthy process (e.g., changing the school's food service vendor, having an adequate teacher:student ratio for physical education classes) or because of budget constraints. By the end of the year, all schools had developed policies, defined problems, and developed language to support healthy eating and physical activity.

**Schools 1 and 2**

Two schools in one district established one SHI team for both schools and collaborated to implement a hand washing policy and a healthy snack policy. The hand washing policy was incorporated into the school handbooks, so the handbooks now state that all children will wash their hands before participating in any activity that involves food. The policy on healthy snacks in all school venues evolved from the district food service director's success in forging relationships with vendors to provide healthy snacks to all elementary schools. The healthy snacks policy was incorporated into the state's mandated school improvement plan (23), giving the team's work greater visibility and acceptance.

**School 3**

School 3 had a low morale issue, so its team had to build support while proposing activities to improve the school's environment. The team drafted polices to replace the less healthy foods that were sold during lunch time to raise money for school events, such as replacing high-fat ice cream with lower fat yogurt and 100% fruit juice popsicles. In addition, the parent–teacher organization attempted to replace fundraisers involving food with little or no nutritional value with fundraisers involving nonfood products. These proposals did not become policies. Sources from the school reported that school profits generated from ice cream sales were considered a needed fundraising strategy and were unlikely to be changed. However, the school's SHI team increased awareness about food choices at special events.

Because of this team's setbacks, facilitators worked with team members to draft a new policy. The policy stated that at the start of each new school year, one SHI team member would collect information from teachers and staff members about the use of curricula or programs that taught children about healthy eating and physical activity. The collected information would be printed in the parent–teacher organization newsletter and school department publications. Although the drafting of this policy unified the team and gave members a sense of accomplishment, the draft did not become a formal policy and no additional progress was made. Despite the team's resourcefulness in developing health promotion policies, enthusiasm for *Eat Healthy and Get Active!* waned after attempts to implement changes were thwarted.

**School 4**

School 4 encountered insurmountable barriers to implementing policies, including personnel changes (replacement of the food service manager) and the second-semester announcement that the school was slated to be closed in the near future. Initially, this school was enthusiastic about *Eat Healthy and Get Active!* After completing the SHI assessment, the team generated and prioritized recommendations. Because the year for the school closing was uncertain, the team decided not to address issues relating to the school's physical structure. Instead, the team drafted three policies. The first stated that the school would try to garner resources through grants and fundraisers for equipment and supplies to increase physical activity and improve physical education. The second stated that students and families would receive health information through after-school programs, school workshops, and other school programs. The third stated that teachers and administrators would encourage student participation in physical activity programs in the community. The announcement that the school would close in June deflated the team's efforts, and the school was unable to implement policies generated by the SHI assessment.

**External facilitators**

External facilitators were an essential component of *Eat Healthy and Get Active!* They worked with schools to establish teams, develop action plans, and monitor progress. In addition, facilitators trained school personnel to use the SHI manual, attended SHI team meetings, drafted agendas, took notes, linked teams with resources in the community, and provided technical assistance. When problems arose, the facilitators played a key role in maintaining the teams' focus so that results of the SHI assessments were translated into action plans and policy recommendations.

**Outcome evaluation**

All schools completed baseline assessments for the three SHI modules. Three schools received high scores for the physical education programs module. Two schools also received high scores for the nutrition services module. No school scored high on the school policies and environment module.

By June 2003, school 3 had completed all three SHI self-assessment modules. Schools 1 and 2 were unable to complete the physical education programs module because of staff turnover. Staff turnover also hampered efforts to complete end-of-study assessments in school 4, a barrier compounded by time constraints caused by the school closing. In school 3, the negative change score from baseline to the end of the study in the nutrition services module was a result of barriers faced by team members as they attempted to translate their recommendations into approved policies (Table 4). In-depth interviews with two team members revealed that proposals for healthy food choices needed greater exposure to gain acceptance and overcome school personnel's reluctance to lose needed revenue from the sale of nonnutritious à la carte and fast-food offerings.

## Discussion

Our needs assessment of 102 elementary schools and interventions in four inner-city elementary schools were undertaken at a time when the prevalence of obesity among Rhode Island children and adolescents was more than double the *A Healthier Rhode Island by 2010* target prevalence of 10% (24). In 2001, approximately 28,612 Rhode Island youth were obese (BMI ≥95th percentile), an overall prevalence rate of 21.5% (25).

One of the challenges in gaining support from school administrators for policy and environmental changes is the absence of data to support the rationale for systems-level nutrition and physical activity interventions. Assessment of policy adoption in school settings is a new area (26); we believe that RINAT is a valuable tool for collecting information on nutrition and physical activity policies and programs in elementary schools and can be modified for middle and high schools.

Findings from RINAT demonstrated that schools with high minority enrollment had fewer nutrition and physical activity programs than schools with low minority enrollment and lacked the infrastructure to promote physical activity, such as outdoor tracks and walking paths. These problems were exacerbated in high minority schools situated in racially segregated neighborhoods. However, our study included only a small sample of racially segregated neighborhoods with high minority schools, and the issue of whether a neighborhood's racial and ethnic composition and socioeconomic status influence a school's physical activity and nutrition environment deserves additional study (4). However, regardless of the racial and ethnic composition of the student body, we found that 85% of elementary schools sold items such as soft drinks, chips, candy, and fast food. All schools need to have sufficient financial resources to support activities such as field trips and school enhancement projects or they will continue to rely on the sale of nonnutritious food items in school venues and at fundraisers to increase revenue and meet budgetary requirements.

We did not find that schools with high minority enrollment were less likely than schools with low minority enrollment to perceive childhood obesity as an important problem. Among principals who reported that childhood obesity was a problem, 47% were in low minority schools and 53% were in high minority schools (*P* = .08; data not shown).

During a 10-month school year, four inner-city Rhode Island elementary schools assessed their school environments and developed action plans using the CDC's SHI. Despite intense pressure to focus all efforts on improving students' reading and math scores and limited resources to dedicate to the intervention project, all four schools completed three of the SHI modules and proposed policies to correct identified problems. Findings from our process evaluation support evaluation results reported by Staten et al (15) on implementing the SHI in low-income schools serving primarily Hispanic students. Like Staten et al (15), we found that implementation of the SHI is less successful in schools with low staff morale, budgetary constraints, academic pressures, and inconsistent administrator support. Also like Staten et al (15), we found that external facilitators were the key to successful policy interventions. With the pressure to focus on reading and mathematics test scores, it is easy for school teams working on nutrition and physical activity policies to lose momentum. Being part of a team that creates policy recommendations is difficult for administrators, teachers, and parents, especially when they are not knowledgeable about national guidelines for healthy eating and physical activity. An outside facilitator keeps the team on track. External facilitators also provide continuity by helping teams overcome barriers such as staff turnover and limited resources.

The process evaluation underscored that the principal must understand the SHI intervention and the school's expectations before agreeing to begin the project. Furthermore, each team member needs to understand the SHI project objectives and expected outcomes. In schools 1 and 2, the drafting and implementation of the hand washing and healthy snacks in school policies was a testament to the essence of an SHI team — collaboration. The school superintendent supported the teams' efforts and considered their accomplishments to be a model for all schools. A 2005 interview with the school principal confirmed that both policies continue to be implemented and are widely accepted by children and parents.

One unique finding from our study was the discovery that although policy interventions for nutrition and physical activity often positively influence student behaviors, hands-on, interactive programs and activities incorporated into policy interventions help schools begin to understand the relationships among policies, behavior change, and a healthy school environment. Including an individual-level behavior change component in a policy intervention provides explicit reinforcement for an SHI team promoting broad-reaching policies that affect all students (12).

Our study has some limitations. Our definition of a high minority school reflected the demographics of Rhode Island and may not be applicable to other states. In addition, our intervention was limited to four schools and three teams. Although many of our findings were similar to those reported by Staten et al (15), the changes we documented may not be generalizable to all elementary schools. We also lacked the funding to evaluate our intervention using a quasi-experimental study design. Administering our needs assessment survey after the intervention was complete would have enabled us to gain a better understanding of factors that influence participation in the SHI process. A postintervention survey also would have provided an opportunity to compare the SHI with other strategies schools use to gain support for healthy eating and physical activity policies. Finally, funding for *Eat Healthy and Get Active!* was only available for one school year. Changing a school's nutritional and physical activity environment is a long-term process. A 1-year intervention is too short to create a sustainable infrastructure.

Our study confirms that the SHI is an effective way to help schools set policies and standards that meet national health objectives. Although our intervention was short term, we believe that the school teams established as part of our intervention will build on their accomplishments and recommend more controversial and high-impact policies. The political climate in Rhode Island is becoming more receptive to the concept of policy and environmental changes to promote healthy eating and physical activity in schools. Obtaining district and statewide support for these policies will ensure long-term implementation of these changes.

## Figures and Tables

**Table 1:** Socioeconomic Characteristics of Students in Participating Elementary Schools, 2001–2002 Rhode Island Needs Assessment Tool

**Characteristics**	**All Schools,** **No. (% Yes)**	**Minority Student Enrollment^a^**		***P *Value** **(*χ* ^2^_1_)**

		**Low,** ** No. (% Yes)**	**High,** ** No. (% Yes)**	

**Individual **				

≥34% of students eligible for free or reduced-price lunch	50 (49.0)	11 (22.0)	39 (78.0)	<.001 (51.7)
Parent education: ≥5% did not complete high school^b^	23 (25.8)	7 (30.4)	16 (69.6)	.009 (6.9)

**Neighborhood **				

Neighborhood with residential racial segregation (≥10% black, ≥15% Hispanic, or both)	12 (11.8)	5 (41.7)	7 (58.3)	.23 (1.5)

**Total**				

Total number of schools	102 (100.0)	59 (57.8)	43 (42.2)	

a Minority student enrollment — *low:* <10% black students and <25% Hispanic students; *high:* ≥10% black students, ≥25% Hispanic students, or both. Data linked to 2000 U.S. census.

b Denominator is 89 schools because of missing data.

**Table 2:** Nutritional and Physical Activity Environments of Participating Elementary Schools, 2001–2002 Rhode Island Needs Assessment Tool

**Variables**	**All Schools** **No. (% Yes)**	**Minority Student Enrollment^a^**		***P *Value** **(*χ* ^2^_1_)**

		**Low** ** No. (% Yes)**	**High** ** No. (% Yes)**	

**Nutrition**				

School has programs for healthy eating^b^	44 (43.1)	32 (54.2)	12 (27.9)^c^	.008 (7.0)
Students can buy nonnutritious foods and sweetened beverages^c^	87 (85.3)	51 (86.4)	36 (83.7)	.70 (0.1)

**Physical activity**				

School has programs for physical activity^d^	72 (70.6)	46 (78.0)	26 (60.5)	.05 (3.7)
School has playground	80 (78.4)	50 (84.7)	30 (69.8)	.07 (3.3)
School has playing field	74 (72.5)	53 (89.8)	21 (48.8)	<.001 (21.0)
School has a track	16 (15.7)	13 (22.4)	3 (7.0)	.04 (4.4)
Students receive 60 minutes or more of physical education per weeke, f	57 (57.0)	33 (57.9)	24 (55.8)	.84 (0.04)
Students participate in 20 minutes or more of recess per day^f^	62 (62.0)	46 (80.7)	16 (37.2)	<.001 (19.7)

a Minority student enrollment — low: <10% black students and <25% Hispanic students; high: ≥10% black students, ≥25% Hispanic students, or both.

b School offers one or more programs to promote healthy eating more than once during the school year or every school year.

c Students can buy snacks that are high fat, high sugar, or both (e.g., cookies, chips, candy), high-calorie fast foods (e.g., french fries, hamburgers, pizza), or sweetened beverages (e.g., soft drinks, fruit drinks that are not 100% fruit juice) during regular school hours in the cafeteria or vending machines or at fundraisers.

d School offers one or more programs to promote physical activity more than once during the school year or every school year.

e Physical education was defined as the number of average minutes each week that a school provided structured physical education classes or lessons, excluding recess.

f Sample based on 100 elementary schools.

**Table 3:** Demographic Characteristics of Participating Intervention Schools in Rhode Island’s 2002–2003 *Eat Healthy and Get Active!* Project

**School Location**	**Grades**	**No. Students**	**% Hispanic**	**% Receiving Free or Reduced-Price Lunch**
School 1: Central Falls school district	Prekindergarten-kindergarten	207	59	93
School 2: Central Falls school district	Kindergarten-1	118	65	83
School 3: Providence school district	Kindergarten-6	547	26	54
School 4: Pawtucket school district	4-6	178	42	88

**Table 4:** Outcome Evaluation Findings for Intervention Schools Participating in Rhode Island’s 2002–2003 *Eat Healthy and Get Active!* Project

**Intervention Schools**	**No. Team Members**	**% Change From Baseline (T1) to End of Study (T2)**			**No. Activities**	**No. Policies Developed**	**No. Policies Implemented**

		**School Policies and Environment** **(T2−T1)**	**Physical Education and Other Physical Activity Programs^c^** **(T2−T1)**	**Nutrition Services^c^** **(T2−T1)**			
Schools 1 and 2: Central Falls school district^b^	6	32.5	…	6.7	5	1	1
School 3: Providence school district	18	5.2	4.5%	**−**7.6	18	1	0
School 4: Pawtucket school district	5	5.5	…	…	4	3	0

a Outcome evaluation findings are based on baseline to end-of-study changes on School Health Index (SHI) self-assessment scores for modules 1, 3, and 4 (www.cdc.gov/HealthyYouth/SHI).

b The two elementary schools in the Central Falls district established one SHI team for both schools.

c Ellipses indicate that end-of-study assessments were incomplete because of barriers encountered by SHI teams.

## Appendix Rhode Island Needs Assessment Tool (RINAT)

**Rhode Island Department of HealthInitiative for a Healthy Weight ProgramSeptember 2001**

[INTERVIEWER INSTRUCTIONS IN CAPS] Survey #_____ Date: ___/___/01 Start time: _______ End time: ________ Interviewer name: ____________________

Thank you for taking the time to talk with me today. As you know from our cover letter, the purpose of the needs assessment is to collect information from elementary school principals about nutrition and physical activity programs and policies in Rhode Island elementary schools. All responses will be pooled and reported for the state as a whole. Individual school responses will not be identified in presentations and reports. The needs assessment is for planning purposes only and is funded by the Centers for Disease Control and Prevention.

If I come to any question that you do not feel comfortable answering, please let me know the name and number of the person you feel I should contact.

Are you ready to begin?

1. [FILL IN]  Current position: ____________________________
  1b. How many years have you been in this position? ________ years

**The next questions ask about nutrition programs at your school *not* counting health education classes.**

2. *Excluding health education classes,* how many programs to promote healthy eating does your school offer regularly? By regularly, I mean *more than once* during the school year or *every* school year. These programs can be given before, during, or after school, such as community gardening, cooking demonstrations, or nutrition programs like Team Nutrition, Bright Start, or Days of Taste. 

0→GO TO 9

___ [FILL IN NUMBER]

3. [IF ONLY 1 PROGRAM] Please tell me the name of the program. 

___________________________________________________ 

[IF MORE THAN 1 PROGRAM] Which nutrition program would you say is your best example of teaching children about healthy eating? __________________________

4. What are the program goals?

1.

2.

3.

5. Is the program offered during or outside of regular school hours?

**_ 1_** □During	**_2_** □Outside	**_3_** □Both	**_8_** □Don't know

6. How often is it offered during the school year?         ____________

7. Who participates in the program — the whole school, or a specific group?

**_ 1_** □Whole school	**_2_** □Specific group	**_3_** □Other__________	**_8_** □Don't know

(Specific grade, special needs)  (Parents/guardians)

8. Does the program have components tailored to children of different racial or ethnic backgrounds — for example, bilingual materials?

**_ 1_** □Yes→[GO TO 8b	**_2_** □No→[GO TO 9	**_8_** □Don't know→GO TO 9

8b. Please describe:._________________________________________

**The next questions ask about physical activity programs at your school *not* counting physical education classes.**

*9. Excluding physical education classes,* how many physical activity programs does your school offer regularly? By regularly I mean *more than once* during the school year or *every* school year. The programs can be given before, during, or after school, such as recreational clubs, sports teams, field days, or Walk Your Kids to School Day.

0→[GO TO 18 IF NO PHYSICAL ACTIVITY PROGRAM AND NO NUTRITION PROGRAM.]

→[GO TO 16 IF NUTRITION PROGRAM BUT NO PHYSICAL ACTIVITY PROGRAM.]

___ [FILL IN NUMBER]

10. [IF ONLY 1 PROGRAM] Please tell me the name of the program. ________________

[IF MORE THAN 1 PROGRAM] Which physical activity program would you say is your best example of teaching children to be physically active?

__________________________

11. What are the program goals?

1.

2.

3.

12. Is the program offered during or outside of regular school hours?

**Table:**

**_ 1_** □During	**_2_** □Outside	**_3_** □Both	**_8_** □Don't know

13. How often is it offered during the school year? ____________ times

14. Who participates in the program — the whole school, or a specific group?

**_ 1_** □Whole school	**_2_** □Specific group	**_3_** □Other__________	**_8_** □Don't know

(Specific grade, special needs)  (Parents/guardians)

15. Do the programs have components tailored to children of different racial or ethnic backgrounds — for example, bilingual materials?

**_ 1_** □Yes→GO TO 15b	**_2_** □No→GO TO 16	**_8_** □Don't know→GO TO 16

15b. Please describe:_________________________________________

**The next three questions ask about the nutrition *and* physical activity programs we just talked about …**

16. Do these programs promote any of the following messages? I'll read some choices:

 a. Eat five or more servings of fruits and vegetables a day?

**_ 1_** □Yes	**_2_** □No	**_8_** □Don't know

b. Eat foods high in calcium?

**_ 1_** □Yes	**_2_** □No	**_8_** □Don't know

c. Be physically active for 30 minutes or more every day?

**_ 1_** □Yes	**_2_** □No	**_8_** □Don't know

d. Any other messages? [LIST] ______________________ 

17. Do the *programs we just talked about* partner with other schools, community programs, or businesses?

**_ 1_** □Yes→GO TO 17b	**_2_** □No→GO TO 18	**_8_** □Don't know→GO TO 18

17b. Please tell me the partners.

Nutrition__________________________

Physical activity____________________

 18. The next questions ask about students' eating habits. Do students have… [READ CHOICES]

a. At least 10 minutes to eat breakfast *once they are seated?*

**_ 1_** □Yes	**_2_** □No	**_8_** □Don't know

b. At least 20 minutes to eat lunch *once they are seated?*

**_ 1_** □Yes	**_2_** □No	**_8_** □Don't know

19. Can students buy the following foods or beverages during regular school hours? This could be in the *cafeteria, vending machines,* or *at fundraisers*. I'll read some choices:

a. Candy?

**_ 1_** □Yes	**_2_** □No	**_8_** □Don't know

b. High-fat snacks? This includes cookies, chips, and ice cream.

**_ 1_** □Yes	**_2_** □No	**_8_** □Don't know

c. High-calorie fast foods? This includes french fries, hamburgers, and pizza.

**_ 1_** □Yes	**_2_** □No	**_8_** □Don't know

d. Sodas, sports drinks, or fruit drinks that are not 100% juice?

**_ 1_** □Yes	**_2_** □No	**_8_** □Don't know

20. Do Coca-Cola, Pepsi-Cola, Dr. Pepper, or other soft drink companies have exclusive rights to sell soft drinks at your school?

**_ 1_** □Yes→GO TO 21	**_2_** □No→GO TO 24	**_8_** □Don't know→GO TO 24

21. Does your school receive a specified percentage of the soft drink sales receipts?

**_ 1_** □Yes	**_2_** □No	**_8_** □Don't know

22. Does your school receive incentives, such as cash awards or donations of equipment, supplies, or other donations, once receipts total a specific amount?

**_1_** □Yes	**_2_** □No	**_8_** □Don't know

23. Is the soft drink bottler allowed to advertise…[READ CHOICES]

a. In the school building?

**_ 1_** □Yes	**_2_** □No	**_8_** □Don't know

b. On the school grounds — for example, outside of the school building, on playing fields?

**_ 1_** □Yes	**_2_** □No	**_8_** □Don't know

c. On school buses?

**_ 1_** □Yes	**_2_** □No	**_8_** □Don't know

**The next questions ask about your school's environment for physical activity.**

24. What percentage of children in your school live within a mile of the school? Your best estimate is fine.

____%

**_ 8_** □Don't know

25. What percentage of children walk to school? Your best estimate is fine.

____%

**_ 8_** □Don't know

26. What do you think prevents children from walking to school who live within a mile of your school? I'll read some choices.

a. Lack of adult supervision, including crossing guards?

**_ 1_** □Yes	**_2_** □No	**_8_** □Don't know

b. No sidewalks or poorly maintained sidewalks?

**_ 1_** □Yes	**_2_** □No	**_8_** □Don't know

c. Neighborhood not safe — for example, crime, neighborhood bullies, dogs roaming street?

**_ 1_** □Yes	**_2_** □No	**_8_** □Don't know

d. Something else I didn't mention? [LIST] ________________________________________________

e. What do you think is the *main barrier* that prevents children from walking to school? ________________________________________________

27. On average, how many minutes *each week* do students spend in physical education classes? Your best estimate is fine. [PROMPT IF NECESSARY. "By physical education, I mean structured physical education classes or lessons, *not recess.*"]

____minutes

**_ 8_** □Don't know

Comments:________________________________________________

28. On average, how many minutes *each day* do students spend in recess? Your best estimate is fine.

____minutes

**_ 8_** □ Don't know

Comments:______________________________________________

29. How crowded are your physical education classes? Would you say very crowded, somewhat crowded, or not crowded at all?

**_ 1_** □Very	**_2_** □Somewhat	**_3_** □Not at all	**_8_** □Don't know

30. Does your school have a gymnasium or a multipurpose room for physical activity?

**_ 1_** □Yes→GO TO 30b	**_2_** □No→GO TO 31	**_8_** □Don't know→GO TO 31

30b. Is the gym/multipurpose room available outside of regular school hours for physical activity?

**_ 1_** □Yes→GO TO 30c, 30d	**_2_** □No→GO TO 31	**_8_** □Don't know→GO TO 31

30c. Is the gym/multipurpose room supervised outside of regular school hours? 

**_ 1_** □Yes	**_2_** □No	**_8_** □Don't know

30d. Is the gym/multipurpose room accessible to children with special needs during the time it is open?

**_ 1_** □Yes	**_2_** □No	**_8_** □Don't know

31. Does your school have a playing field?

**_ 1_** □Yes→GO TO 31b	**_2_** □No→GO TO 32	**_8_** □Don't know→GO TO 32

31b. Is the playing field available outside of regular school hours?

**_ 1_** □Yes	**_2_** □No	**_8_** □Don't know

32. Does your school have a playground?

**_ 1_** □Yes→GO TO 32b	**_2_** □No→GO TO 33	**_8_** □Don't know→GO TO 33

32b. Is the playground available outside of regular school hours?

**_ 1_** □Yes	**_2_** □No	**_8_** □Don't know

33. Does your school have a walking or running track or fitness trail?

**_ 1_** □Yes	**_2_** □No	**_8_** □Don't know

**The next questions ask about written nutrition and/or physical activity policies at your school.**

*34. Excluding nutrition and physical activity policies mandated by the state,* does your school have its own written policies on nutrition and/or physical activity?

**_ 1_** □Yes→GO TO 34a-34h	**_2_** □No→GO TO 36	**_8_** □Don't know→GO TO 36

Do you have … [READ CHOICES]

a. A *written policy* for serving healthy school meals that are low in fat, sodium, and added sugars?

**_ 1_** □Yes	**_2_** □No	**_8_** □Don't know

b. A *written policy* for prohibiting the sale of candy, chips, soft drinks and other foods of low nutritional value …

- (1) During school meals?
  **_ 1_** □Yes	**_2_** □No	**_8_** □Don't know
- (2) At school events including fundraisers?
  **_ 1_** □Yes	**_2_** □No	**_8_** □Don't know

c. A *written policy* for prohibiting the use of food as a reward or punishment?

**_ 1_** □Yes	**_2_** □No	**_8_** □Don't know

d. A *written policy* on how physical and health education are scheduled, aside from the state mandate of providing 100 minutes per week?

**_ 1_** □Yes	**_2_** □No	**_8_** □Don't know

e. A *written policy* on how nutrition education is provided *within or outside of* the health education curriculum?

**_ 1_** □Yes	**_2_** □No	**_8_** □Don't know

f. A *written policy* for providing daily recess for all grades?

**_ 1_** □Yes	**_2_** □No	**_8_** □Don't know

g. A *written policy* for supporting walking and/or biking to school?

**_ 1_** □Yes	**_2_** □No	**_8_** □Don't know

h. A *written policy* for prohibiting the use of physical activity as punishment?

**_ 1_** □Yes	**_2_** □No	**_8_** □Don't know

35. In general, how often are your written policies enforced? Would you say all of the time, often, some of the time, or not at all?

**_ 1_** □All	**_21_** □Often	**_3_** □Some	**_4_** □Not at all	**_8_** □Don't know

36. Are families involved in making recommendations for… [READ CHOICES]

a. School food service meals?

**_ 1_** □Yes	**_2_** □No	**_8_** □Don't know

b. Nutrition programs?

**_ 1_** □Yes	**_2_** □No	**_8_** □Don't know

c. Physical activity programs?

**_ 1_** □Yes	**_2_** □No	**_8_** □Don't know

d. School policies related to nutrition and/or physical activity?

**_ 1_** □Yes	**_2_** □No	**_8_** □Don't know

**The next questions ask about data.**

37. Do you collect student information on …[READ CHOICES]

a. Demographics (age, gender, race/ethnicity)?

**_ 1_** □Yes	**_2_** □No	**_8_** □Don't know

↓

(1) Is the information computerized?

**_ 1_** □Yes	**_2_** □No	**_8_** □Don't know

(2) Who collects it?_____________________________

b. Height and weight?

**_ 1_** □Yes	**_2_** □No	**_8_** □Don't know

↓

(1) Is the information computerized?

**_ 1_** □Yes	**_2_** □No	**_8_** □Don't know

(2) Who collects it?_____________________________

c. Scores on standardized physical fitness tests?

**_ 1_** □Yes	**_2_** □No	**_8_** □Don't know

↓

(1) Is the information computerized?

**_ 1_** □Yes	**_2_** □No	**_8_** □Don't know

(2) Who collects it?_____________________________

**The last questions ask for your opinions.**

38. Do you think that overweight or obesity is a problem or a potential problem among children in your school?

**_ 1_** □Yes (problem now)	**_2_** □Yes (potential problem)	**_3_** □No	**_8_** □Don't know

39. If you had the school resources, how would you *increase healthy eating* among children in your school?

40. If you had the school resources, how would you *increase physical activity* among children in your school?

If you have any of the following materials, please fax them today to [INSERT CONTACT NAME AND TELEPHONE NUMBER].

[READ CHOICES. MARK ALL THAT APPLY.]

[ ] Flyers or descriptions of your school's nutrition and/or physical activity programs

[ ] Written policies for nutrition and/or physical activity

[ ] Sample data collection forms for student information (e.g., demographics [age, gender, race/ethnicity], height and weight, physical fitness test scores)

**[IF ADDITIONAL INFORMATION NEEDED TO COMPLETE SURVEY.]**

Can you suggest a day and time that we may contact you again to obtain additional information needed?

Day:_________________       Time: ______ am ______ pm

Thank you for again for your time. If you have any questions about the needs assessment tool or our project, please contact [INSERT CONTACT NAME, TELEPHONE NUMBER, AND ADDRESS]. Our staff will be happy to send you a copy of the results.

**INTERVIEWERS: IF RINAT IS SELF-ADMINISTERED BY SCHOOL, DO NOT FAX THIS PAGE. FILL OUT BEFORE GIVING COMPLETED RINAT TO **[INSERT CONTACT NAME].

Interview day and time ___________

Interview reschedule: date #1 ___________

Interview reschedule: date #2 ___________

Interview complete: date ___________

Forms received? (yes/no) ___________

[ONE FOLLOW-UP PHONE CALL ONLY]

Follow-up call: date ___________ 
